# Role of Different Low-Density Lipoprotein-Lowering Medications on Secondary Prevention of Atherosclerotic Cardiovascular Disease in Patients With Diabetes Mellitus

**DOI:** 10.7759/cureus.40905

**Published:** 2023-06-24

**Authors:** Jordan L Saag, Dennis Gross, Daniel Stirt, Andrea Espina Rey, Bernard Gros

**Affiliations:** 1 Medical School, University of Central Florida College of Medicine, Orlando, USA; 2 Epidemiology and Public Health, University of Central Florida College of Medicine, Orlando, USA; 3 Internal Medicine, University of Central Florida College of Medicine, Orlando, USA

**Keywords:** cholesterol-lowering medications, secondary prevention, diabetes mellitus, atherosclerotic cardiovascular disease, hypercholesterolemia

## Abstract

Purpose

The objective of this study was to explore the optimal cholesterol-lowering therapy for diabetic patients categorized as having a very high risk for future atherosclerotic cardiovascular disease (ASCVD) events. The primary medications under investigation were statins, ezetimibe, and proprotein convertase subtilisin-kexin type 9 (PCSK9) inhibitors (PCSK9-Is). The efficacy of different medication regimens helped to draw conclusions regarding the evolution of cholesterol management recommended under the American College of Cardiology's (ACC) 2013 and 2018 guidelines.

Methods

A retrospective chart review was conducted on a cohort of patients from a large, community-based cardiology practice. Inclusion criteria specified patients aged 30-82 with a past medical history of two or more ASCVD events or one ASCVD event and at least two high-risk comorbidities. Acquired data included demographics, all lipid panels, medications used, and ASCVD events between December 1, 2013, and December 31, 2019. The data were stored and encrypted on a REDCap account. Sub-group analysis was conducted on only diabetic patients, who were then categorized by medication regimen. The statistical analysis was completed using Fisher’s exact test. A p-value <0.05 was considered significant.

Results

A total of 102 diabetic patients met the inclusion criteria. Our primary analysis determined the percentage of patients who achieved their goals on each medication regimen. The goal was defined as a low-density lipoprotein cholesterol (LDL-C) level of less than 70 mg/dL or at least a 50% reduction from baseline levels. The results are as follows: none (0%), statin (33.9%), ezetimibe (21.1%), statin + ezetimibe (73.5%), PCSK9-Is ± statin (83.3%), and PCSK9-Is and ezetimibe ± statin (100%). There proved to be a significant difference favoring all combination regimens over statins alone; however, there was no significant difference between these advanced regimens. A follow-up analysis determined if these patients were able to maintain their goals in the subsequent lipid panel after achieving their goals. The results are as follows: none (0%), statin (61.5%), ezetimibe (50%), statin + ezetimibe (77.8%), PCSK9-Is ± statin (100%), and PCSK9-Is and ezetimibe ± statin (66.6%). The only significant difference found was between PCSK9-Is ± statins and statins alone.

Conclusions

Our study revealed that regimens using PCSK9 inhibitors and ezetimibe, in addition to maximally tolerated statin therapy, were more effective than statin therapy alone in achieving the goal. On extended analysis, only PCSK9 inhibitors showed superior ability in terms of maintaining the goals for diabetic patients at very high risk for future ASCVD events. This implies that statins alone may be inadequate to properly treat this specific patient population. In the context of clinical practice, physicians could have heightened consideration for dual therapy consisting of maximally tolerated statins and a secondary agent in accordance with the 2018 ACC guidelines.

## Introduction

Heart disease is the leading cause of death among men and women in the United States today [1]. Atherosclerotic cardiovascular disease (ASCVD) is one of the significant contributors to this trend. Hypercholesterolemia, defined as elevated levels of low-density lipoprotein cholesterol (LDL-C), is a direct risk factor for ASCVD [2-4]. For this reason, the management of LDL-C levels has been integral to the management of ASCVD and the primary and secondary prevention of ASCVD events, such as myocardial infarction (MI) and stroke. Maintaining low LDL-C levels is associated with a significant reduction in the risk of these events [5].

The American College of Cardiology (ACC) and American Heart Association (AHA) released the 2013 ACC/AHA Guideline on the Treatment of Blood Cholesterol to Reduce Atherosclerotic Cardiovascular Risk in Adults to improve cholesterol management for all adult patients [6]. These guidelines recommended maximally tolerated statins as the first-line therapy for women and men under the age of 75 diagnosed with clinical ASCVD. This recommendation also applies to those deemed to be at very high risk for future ASCVD events [6].

In 2018, these guidelines were updated to include PCSK9 inhibitors (PCSK9-Is), such as evolocumab, alirocumab, and ezetimibe [7]. The use of ezetimibe, in addition to a maximally tolerated statin therapy regimen, is now considered a class I recommendation in very high-risk patients with LDL-C greater than 70 mg/dL [7]. The addition of proprotein convertase subtilisin-kexin type 9 (PCSK9) inhibitors (PCSK9-Is) is only indicated after failure of the regimen above to reduce LDL-C levels below 70 mg/dL or non-HDL-C levels below 100 mg/dL [7].

Diabetic patients are at a much greater risk of ASCVD, commonly presenting with obesity, hypercholesterolemia, hypertriglyceridemia, and hypertension [8]. As a result, diabetic patients also display more frequent histories of ASCVD events, and in turn, a larger proportion is labeled "very high-risk for future events" [9]. Thus, it is particularly critical for diabetic patients to maintain lower levels of LDL-C. This results in more frequent and aggressive treatment regimens needed for such patients in clinical practice [9].

This study highlights the challenges of achieving new cholesterol targets in clinical practice and how often practitioners should expect to supplement statin therapy to achieve and maintain goal LDL-C. Understanding these trends can improve medical practice and future guidelines, helping physicians provide optimal care for these comorbid patients.

This study was previously presented as a poster at the 2022 Cardiometabolic Health Conference on October 19, 2022.

## Materials and methods

Study design

A retrospective chart review was performed on a cohort of patients at the Cardiac and Vascular Institute, a large multi-centered community-based cardiology practice in north Florida, between December 1, 2013, and December 31, 2019. Patients were assessed for whether they met guideline goals for ASCVD management under various medication regimens. The goal was defined as an LDL-C value of <70 mg/dL or a >50% reduction in LDL-C from their baseline, based on consecutive lipid panels [7]. Baseline values were established from their most recent lipid panel before the study start date. If no baseline lipid panel was available before the study start date, a panel before September 30, 2015, was used, given that the patient was untreated or only on recommended statin therapy. A minimum of three lipid panels were collected for each patient. Individual patients were then analyzed by medication regimen with the outcomes of achieving and not achieving goals. Medication regimens included: none, statin alone, ezetimibe alone, statin and ezetimibe, PCSK9-Is and maximally tolerated statin therapy (PCSK9-Is ± statin), and PCSK9-Is and ezetimibe and maximally tolerated statin therapy (PCSK9-Is and ezetimibe ± statin). Patients were accounted for multiple times if they achieved goals on different regimens throughout the study period. Maintenance of the goal was defined as achievement on at least two consecutive panels. Patients were excluded from this portion of the analysis if they lacked regimen continuity after initial goal achievement.

Participant selection

Patients included in this study were aged 30-82, had established ASCVD, and were deemed to be at very high risk for future ASCVD events. A very high-risk determination consisted of those with either a history of two previous ASCVD events or only one event and at least two high-risk comorbidities. High-risk comorbidities included age ≥ 65, familial hypercholesteremia (FH), past arterial revascularization, chronic kidney disease (CKD), currently smoking, past coronary artery bypass graft (CABG), past percutaneous coronary intervention (PCI), congestive heart failure (CHF), and diabetes mellitus (type I or II).

Data collection

ASCVD events collected from patient medical charts included stroke, transient ischemic attack (TIA), coronary artery disease (CAD), acute coronary syndrome (ACS), past arterial revascularization, peripheral vascular disease (PVD), and aortic aneurysm. Variables collected on medication regimens included lipid-lowering agent(s), dosage, and duration of use. Patient demographics were also recorded, including age, sex, and race or ethnicity. Study data were collected and managed by Research Electronic Data Capture (REDCap) (Vanderbilt University Medical Center, Nashville, Tennessee, USA) electronic data capture tools hosted at the University of Central Florida College of Medicine, Orlando, USA [10].

Statistical test

All statistical tests used R (version 4.2.3; R Core Team, R Foundation for Statistical Computing, Vienna, Austria) and RStudio (version 2022.07.2; Posit PBC, Boston, Massachusetts, USA). Statistical analyses were only performed on the subgroup of diabetic patients. Descriptive analyses were performed on lipid and medication regimen variables. For inferential analyses, patient lipid panels were classified by concurrent medication regimens during blood draws. A pairwise Fisher’s exact test was then used to compare the efficacy of regimens against one another. Statistical significance was determined as a p-value <0.05.

## Results

A total of 102 diabetic patients met the study criteria. Patient demographics are reported in Table 1.

**Table 1:** Patient demographic information and prevalence of ASCVD events (n=102). *Mean

Characteristic			Value
Sex, n (%)			
	Male		59 (57.8)
	Female		43 (42.2)
Race, n (%)			
	White		82 (80.4)
	Black		13 (12.7)
	Other		7 (6.9)
Age* (range in years)			62.4 (37-76)

ASCVD events recorded are listed in Table 2. The most common ACSVD event observed was CAD (95.1%), followed by arterial revascularization (77.5%) and angina (45.1%).

**Table 2:** ASCVD events in the population (n=102)

Event	Population %
Stroke	2.0
Transient ischemic attack	2.9
Coronary artery disease	95.1
Angina	45.1
Acute coronary syndrome	41.2
Arterial revascularization	77.5
Peripheral vascular disease	22.5
Aortic aneurysm	2.9

**Figure 1:**
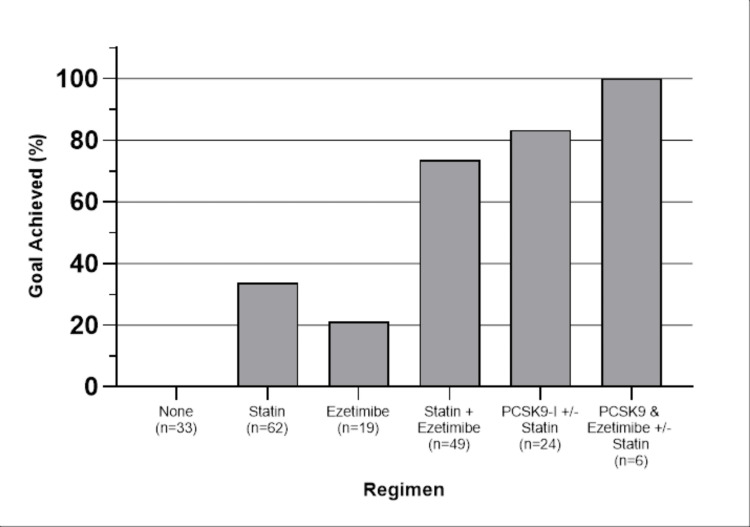
The percentage of patients who achieved the goal

The percentage of patients who achieved their goals on each regimen is shown in Figure 1.

Dual therapy with PCSK9-Is and ezetimibe had the highest efficacy, with 100% of patients achieving their goals. Ezetimibe therapy alone had the lowest efficacy, with only 20% of patients reaching their goals. Since it was possible for patients to achieve goals on multiple different regimens over the duration of the study, the total number of data points collected is 193.

Table 3 displays Fisher’s exact test results for all possible combinations of regimens. Of note, there was a significant difference in the ability to achieve the goal, favoring both statin + ezetimibe and PCSK9-Is combination regimens over statins. There was no significant difference between statin + ezetimibe, and PCSK9-Is in achieving the goal.

**Table 3:** Fisher’s exact test comparing the efficacy of medication regimens in achieving the goals *= p<0.05; **= p<0.01; ***= p<0.001; NS = not significant

Regimen A	Regimen B	Preferred regimen	p-value
None	Statin	B	***
	Ezetimibe	B	*
	Statin + Ezetimibe	B	***
	PCSK9I ± Statin	B	***
	PCSK9I + Ezetimibe ± Statin	B	***
Statin	Ezetimibe	-	NS
	Statin + Ezetimibe	B	***
	PCSK9I ± Statin	B	***
	PCSK9I + Ezetimibe ± Statin	B	**
Ezetimibe	Statin + Ezetimibe	B	***
	PCSK9I ± Statin	B	***
	PCSK9I + Ezetimibe ± Statin	B	**
Statin + Ezetimibe	PCSK9I ± Statin	-	NS
	PCSK9I + Ezetimibe ± Statin	-	NS
PCSK9I ± Statin	PCSK9I + Ezetimibe ± Statin	-	NS

**Figure 2:**
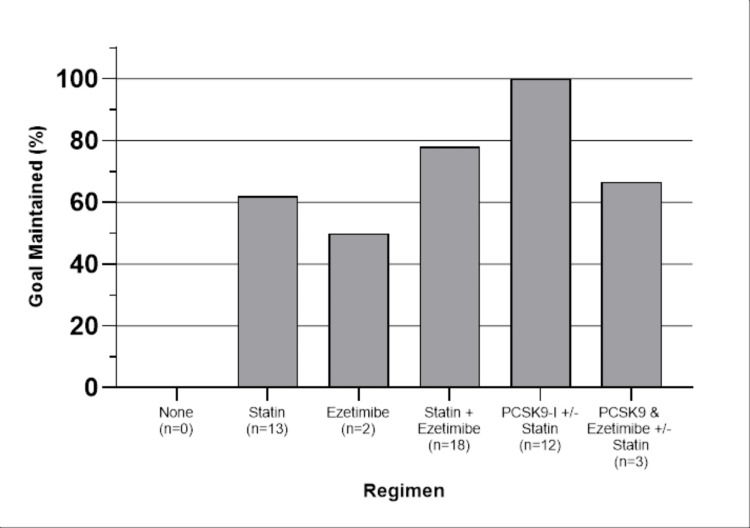
Percentage of patients that maintained their goal after achieving it

The percentage of patients maintaining goals after the achievement is shown in Figure 2.

PCSK9-Is had the highest maintenance rate at 100%, whereas ezetimibe alone had the lowest rate at 50%. Patients were excluded from this portion of the analysis if they did not achieve the goal initially (n=106), or were lost to follow-up, or changed regimens at the subsequent lipid panel (n=39). Forty-eight total data points were remaining. Table 4 displays the results of Fisher’s exact test. The only significant difference seen in goal maintenance was the use of PCSK9-Is ± statins.

**Table 4:** Fisher’s exact test comparing the efficacy of medication regimens in maintaining the goal *= p<0.05; NS: not significant

Regimen A	Regimen B	Preferred regimen	p-value
None	Statin	-	-
	Ezetimibe	-	-
	Statin + Ezetimibe	-	-
	PCSK9I ± Statin	-	-
	PCSK9I + Ezetimibe ± Statin	-	-
Statin	Ezetimibe	-	NS
	Statin + Ezetimibe	-	NS
	PCSK9I ± Statin	B	*
	PCSK9I + Ezetimibe ± Statin	-	NS
Ezetimibe	Statin + Ezetimibe	-	NS
	PCSK9I ± Statin	-	NS
	PCSK9I + Ezetimibe ± Statin	-	NS
Statin + Ezetimibe	PCSK9I ± Statin	-	NS
	PCSK9I + Ezetimibe ± Statin	-	NS
PCSK9I ± Statin	PCSK9I + Ezetimibe ± Statin	-	NS

## Discussion

Our results highlight some key trends in cholesterol management in very high-risk diabetic patients, illustrating the everyday clinical use of different lipid-lowering drugs and how those regimens perform over extended use. Patients on statin therapy alone could only achieve the guideline goals in lipid management 33.9% of the time. This generally improved under dual therapy regimens, with the addition of ezetimibe increasing efficacy to 73.5% and PCSK9-Is improving it to 80%. Between these dual therapy regimens, there was no significant difference in the ability to achieve the goal, with patients on statin + ezetimibe performing comparably to those that used PCSK9-Is ± statin (p=0.395) and PCSK9-Is and ezetimibe ± statin (p=0.317). However, when examining patients that not only achieved but maintained their goals long-term, PCSK9-Is ± statin recorded a 100% maintenance rate, whereas statin + ezetimibe showed no significant difference compared to statins alone (p=0.433).

These findings indicate that statin + ezetimibe or a combination PCSK9-Is therapy is superior to statins alone in managing very high-risk diabetic patients. In this regard, our results align with the advice of the 2018 ACC guidelines and support using these regimens to achieve our goals. This is consistent with previous clinical trials, one of which shows that PCSK9-Is in combination with statin therapy decreased median LDL-C values by 59% compared to placebo and even significantly decreased the risk of adverse cardiovascular events [11]. Our results also demonstrate that PCSK9-Is therapy may provide more consistent cholesterol management in the long term compared to statins + ezetimibe. Multiple factors can explain this. Primarily, PCSK9-Is are known to be extremely potent, with one large clinical trial finding the mean difference in LDL-C reduction between PCSK9-Is and ezetimibe monotherapy to be 30.4% [12]. Another factor could involve the pharmacokinetics of PCSK9-Is and their dosing within the population. PCSK9-Is have an estimated half-life of 11-17 days and are currently given as subcutaneous injections biweekly or monthly [13]. Once a steady state is achieved, this method of administration could reduce the likelihood of medication nonadherence and may promote goal maintenance over time.

An unexpected result of this study was the lack of a significant difference between statin and ezetimibe in achieving the goal (p=0.398). Statin therapy is, after all, the first-line medication of choice, and ezetimibe’s use as monotherapy is typically limited to patients with statin intolerance. Because this study was a retrospective chart review, it is unknown whether statin therapy was fully maximized before adding a second agent. In this scenario, the true power of statins could be misrepresented, as some lipid panels may have been drawn with lower doses.

Our results shed light on the current standards and successes in cholesterol management at a time when treatment guidelines were changing. In addition, our population was drawn from a large community-based practice, allowing for relatively consistent long-term records and a real-world view of cardiology practices. Focusing only on a subset of very high-risk diabetic patients provides insight into their specific medical needs and treatment patterns, with the goal of secondary prevention. As discussed previously, prolonged maintenance of goal LDL-C reduces this risk [5]. Our data suggest that combination regimens, especially those with PCSK9-Is, can aid in consistent control of LDL-C over time and enhance secondary prevention.

It is essential to recognize updates to current guidelines and the advent of new therapies released after the end of our study period. The American College of Cardiology released an expert consensus decision pathway in 2022, lowering the goal LDL-C from 70 mg/dL to 55 mg/dL [14]. This benchmark only supports the need for higher consideration of adjunctive therapies in addition to maximally tolerated statins. Options for supportive treatments are expanding, with drugs like inclisiran, bempedoic acid, evinacumab, and others being approved in similar high-risk populations and patients with familial hypercholesterolemia [14].

Some limitations of our study must be acknowledged. Primarily, many factors play a role in LDL-C values outside of the medication regimen that could affect results. These include diet, exercise level, genetic predisposition, etc. In diabetic patients specifically, certain medications, including metformin, sulfonylureas, and thiazolidinediones, have variable effects on plasma lipids, potentially confounding the results [15]. The methodology of our study must also be considered. In a retrospective chart review, there is no control over the time between subsequent lipid panels and consistent adherence to one medication regimen. During the time period of this study, adherence proved to be a problem for PCSK9-Is due to the enormous expense and rigid pre-authorization process [16]. More research should be done to see how those common problems affect treatment and outcomes in diabetic patients.

## Conclusions

Diabetes mellitus carries a heightened risk of developing ASCVD and its potential sequelae. Those falling under the umbrella of very high risk undoubtedly benefit from strict cholesterol control, the bar for which is trending lower and lower. Our results demonstrate that this population has relatively poor success in achieving and maintaining goal LDL levels with statins alone. Therefore, it is reasonable to consider accelerated use of combination therapy with ezetimibe or PCSK9-Is. The practice of such measures could play an important role in secondary prevention, allowing very high-risk diabetic patients some protection against cardiovascular-related morbidity and mortality.
